# Higher visceral fat area is inversely associated with ketosis in Chinese patients with type 2 diabetes mellitus: a cross-sectional study

**DOI:** 10.3389/fendo.2026.1948929

**Published:** 2026-09-11

**Authors:** Jun-Wei Wang, Le Jiang, Jie Ren, Lin-lin Gao, Ya-Nan Qiao, Shi-Wei Liu

**Affiliations:** 1 Department of Endocrinology, Shanxi Bethune Hospital, Shanxi Academy of Medical Sciences, Third Hospital of Shanxi Medical University, Tongji Shanxi Hospital, The Key Laboratory of Endocrine and Metabolic Diseases of Shanxi Province, Taiyuan, China; 2 Department of Endocrinology, Inner Mongolia Autonomous Region People's Hospital, Hohhot, China; 3 Department of Orthopedics, Shanxi Bethune Hospital, Shanxi Academy of Medical Sciences, Third Hospital of Shanxi Medical University, Tongji Shanxi Hospital, Taiyuan, China; 4 Department of Pharmacy, Second Hospital of Shanxi Medical University, Taiyuan, China

**Keywords:** ketosis, MASLD, type 2 diabetes mellitus, visceral fat area, visceral obesity

## Abstract

**Background:**

The relationship between visceral fat area (VFA) and ketosis remains a subject of debate, with a paucity of research specifically addressing this association in individuals with type 2 diabetes mellitus (T2DM). This research seeks to investigate the connection between VFA and ketosis among Chinese patients diagnosed with T2DM.

**Methods:**

A cross-sectional study was performed, encompassing 1,058 patients with T2DM from real-world clinical environments. Body composition and visceral obesity were assessed through bioelectrical impedance analysis. The clinical characteristics of patients across varying quartiles of VFA were compared, and the relationships between VFA quartiles, visceral obesity, and ketosis were investigated.

**Results:**

After adjusting for age, gender and diabetes duration, ketosis prevalence appeared to gradually decrease across ascending VFA quartiles, with rates of 15.5%, 11.5%, 9.1% and 5.3% respectively, presenting a statistically significant trend (P < 0.001). Higher VFA categories tended to correlate with higher MASLD prevalence and elevated C-peptide levels (P < 0.001 for trend). Multivariate binary logistic regression indicated a potential inverse link between visceral obesity (OR = 0.409, 95%CI: 0.251–0.644, P<0.001), VFA quartile and ketosis occurrence among T2DM patients (P = 0.022 for trend).

**Conclusions:**

Higher visceral fat mass was associated with a lower prevalence of ketosis. Evaluation of visceral fat status can provide reference for personalized diet and medication management. Attention should be paid to ketosis in patients with low visceral fat.

## Introduction

The rise in obesity has turned into a worldwide public health concern, with its occurrence growing consistently across the globe. Visceral obesity is marked by fat accumulation mainly in the abdominal area. Research indicates that after excessive caloric intake, subcutaneous abdominal fat reaches its expansion limit, resulting in the buildup of visceral and ectopic fat (1). A study of over 440,000 individuals revealed a 29.1% prevalence of visceral obesity, with higher rates observed in T2DM patients (2). An investigation using a visceral fat areas(VFA) cutoff value of ≥100 cm² for diagnosing visceral obesity revealed that 39.7% of T2DM patients exhibited visceral obesity, with 11.5% of patients with normal weight also exhibiting visceral obesity (3).

Research indicates that visceral fat is strongly linked to multiple aspects of metabolic syndrome, such as high blood pressure, elevated triglycerides, and ectopic fat storage (4–6). Visceral fat and hyperuricemia had a J-shaped association, with the visceral adiposity index serving as an independent risk factor (5). Furthermore, a rise in visceral fat was linked to more severe inflammation and fibrosis in the liver. Visceral fat served as an independent predictor for the aggravation of steatohepatitis, even when considering insulin resistance and liver fat (7). Furthermore, elevated visceral fat levels were closely linked to insulin resistance (8). In summary, visceral obesity accelerates the progression of various metabolic components.

Ketosis is a state marked by an abnormal buildup of ketone bodies resulting from irregular fat metabolism. Nutritional ketosis, occurring without disease, is usually triggered by alcohol consumption or fasting. In the general population, more visceral fat is connected to an elevated ketosis. The ketogenic diet, which features low carbohydrate, high fat, and moderate protein levels, has gained popularity as a weight loss strategy (9). Several studies reported that individuals with visceral obesity undergoing ketogenic diets experienced reductions in fat mass and visceral fat area, accompanied by significant increases in ketone body levels (10–14). Alternatively, a study demonstrated that people suffering from obesity had reduced levels of ketone bodies compared to the general population (15). In conclusion, the link between visceral fat and ketosis development is still debated.

Diabetic ketosis is a pathological condition characterized by elevated ketone levels. Studies indicate that a 1 mmol/L rise in urinary ketone levels correlates with a 5% increase in mortality among T2DM patients (16). The relationship between obesity and ketosis in T2DM has garnered significant attention. In patients with T2DM, following a ketogenic diet demonstrated to lower insulin levels, which in turn reduced blood glucose, and also effectively decreased waist circumference and body weight (9, 17). Moreover, a study suggested that obesity exacerbated lipid metabolism disorders and insulin resistance in T2DM patients, which was further compounded by elevated blood ketone levels (18). A meta-analysis showed that T2DM patients with a BMI above 31 kg/m² had a higher rate of ketoacidosis than those with a BMI below 31 kg/m² (19). Conversely, a study found that T2DM patients with ketoacidosis tended to have a lower BMI compared to those without ketoacidosis (20). Another study found no notable difference in BMI between ketosis-prone and non-ketosis-prone T2DM patients (21). The link between obesity and ketosis in T2DM is still unclear, with previous research often neglecting the connection between visceral fat measurements and ketosis occurrence in T2DM.

In patients with T2DM, the relationship between ketosis and visceral adiposity remains inadequately characterized. Consequently, this study aims to elucidate the correlation between visceral fat and the occurrence of ketosis in individuals with T2DM, in addition to examining the factors associated with this relationship.

## Methods

### Subjects and study design

This cross-sectional study included adults diagnosed with T2DM who were consecutively admitted to the Department of Endocrinology at Shanxi Bethune Hospital between January and December 2020. Written informed consent was obtained from all participants, and the study was approved by the hospital’s ethics committee (YXLL-2026-110). Eligible participants were adults with T2DM, BMI ≥18.5 kg/m² (non-underweight established by the WHO) (22), and complete VFA, subcutaneous fat areas (SFA) and biochemical marker data (23). Exclusion criteria included recent or current heavy alcohol use, ketogenic or low-carbohydrate diets, restrictive diets, use of sodium-glucose cotransporter-2 (SGLT2) inhibitors, and conditions such as severe systemic or infectious diseases, acetoacetate dehydrogenase deficiency, cirrhosis, severe liver dysfunction, and organophosphate pesticide poisoning. Participants were stratified into quartiles based on VFA, with a target sample size of 1058 eligible T2DM patients for analysis (**Supplementary Figure 1**).

### Clinical assessment and diagnostic evaluations

Upon admission, data were collected on patients’ history of hypertension and dyslipidemia, alcohol and smoking habits, duration of diabetes, use of insulin or its analogs (IIAs), lipid-lowering and oral antidiabetic drugs (LLDs and OADs), as well as anthropometric data (24). Smoking and drinking were classified as current or former. Hypertension was defined as SBP ≥ 140 mmHg, DBP ≥ 90 mmHg, or a previous diagnosis with ongoing treatment. Dyslipidemia was marked by triglyceride (TG) levels of at least 1.7 mmol/L, low-density lipoprotein cholesterol (LDL-C) levels of 3.37 mmol/L or greater, total cholesterol (TC) levels of 5.18 mmol/L or more, high-density lipoprotein cholesterol (HDL-C) levels less than 1.04 mmol/L, or the present use of lipid-lowering drugs (LLDs).

Blood was drawn after fasting overnight on the morning of the second day after being admitted. Laboratory tests, conducted following standardized protocols, included glycosylated lipid profile, hemoglobin A1C (HbA1c), alanine aminotransferase (ALT), serum uric acid (SUA) and serum creatinine (sCr). Participants underwent a 75 g oral glucose tolerance test, with venous blood samples collected at baseline and at 30, 60, and 120 minutes post-glucose challenge. The areas under the curve (AUCs) for plasma glucose and C-peptide were computed using the trapezoidal rule as follows: Glucose AUC = [(fast plasma glucose (FPG) + glucose_30_min)/2 × 30] + [(glucose_30_min + glucose_60_min)/2 × 30] + [(glucose_60_min + glucose_120_min)/2 × 60]; C-peptide AUC = [(fast C-peptide (FCP) + C-peptide_30_min)/2 × 30] + [(C-peptide_30_min + C-peptide_60_min)/2 × 30] + [(C-peptide_60_min + C-peptide_120_min)/2 × 60]. To evaluate insulin resistance, the HOMA2-IR was calculated using the validated HOMA2 calculator from the Diabetes Trials Unit at the University of Oxford. Morning urine samples were collected on the second day to measure urinary ketones and the albumin-to-creatinine ratio in urine (uACR). Urinary ketones were assessed using the nitroprusside method. Ketosis is characterized by urinary ketones (≥2+) alongside a plasma glucose level of at least 11.1 mmol/L (25). Diabetic ketoacidosis (DKA) was diagnosed based on blood gas analysis, using a cutoff of pH below 7.35.

### Bioelectrical impedance analysis and abdominal ultrasonography

VFA and SFA were measured using the OMRON DUALSCAN HDS-2000 (Kyoto, Japan) after a minimum 12-hour fast. Participants lay supine with wrists, ankles, and the umbilicus exposed. Following voiding and quiet rest, umbilical cross-sectional dimensions were obtained with participants breath-holding at end-expiration. Dual-frequency bioelectrical impedance analysis was performed via an abdominal electrode belt and limb electrodes. A certified physician conducted all measurements per the manufacturer’s protocol (**Supplementary Figure 2**). Visceral obesity was defined as VFA ≥ 80 cm² according to the diagnostic criteria applicable to Chinese population (26).

Experienced ultrasonographers conducted abdominal ultrasonography using a Philips EPIQ 7 system with a 3.5 MHz convex transducer, without access to laboratory results. MASLD was diagnosed after excluding other hepatic conditions, such as excessive alcohol intake and viral hepatitis, based on ultrasonographic evidence of hepatic steatosis. The diagnosis necessitated at least two features: increased hepatic echogenicity compared to kidney or spleen, blurring of intrahepatic vessels, and reduced ultrasound penetration, alongside a confirmed T2DM diagnosis (27).

### Statistical analysis

Data analysis was conducted using the full-functional 14-day trial version of IBM SPSS Statistics 32.0. The Kolmogorov–Smirnov test checked normality of continuous variables. Data that follow a normal distribution are presented as mean ± standard deviation, while non-normal data are shown as median with interquartile range. For comparing two groups, either a t-test or Mann–Whitney U test was utilized, whereas one-way ANOVA or Kruskal–Wallis H test was applied for multiple group comparisons. Frequencies and percentages were used to present categorical variables, which were analyzed using the chi-square test. Binary logistic regression was used for categorical outcomes and univariate linear regression for continuous outcomes, adjusting for sex, age, and DD. Binary logistic regression analysis was performed to examine the association between VFA quartiles and ketosis, as well as the association between visceral obesity and ketosis. Collinearity diagnostics were performed to calculate VIFs, with a threshold of <5 indicating no significant multicollinearity. Five models were developed with varying levels of adjustment: Model 1 was unadjusted; Model 2 included adjustments for age, sex, DD, smoking, alcohol consumption, dyslipidemia, and hypertension; Model 3 added adjustments for LLDs, IIAs, and OADs; Model 4 incorporated further adjustments for SBP, DBP, BMI, and SFA; Model 5 was defined as a sensitivity analysis with additional adjustments for lipid profile, sCr, SUA, uACR, ALT, HbA1c, HOMA2-IR, glucose AUC, and C-peptide AUC. This model has a low events-per-variable ratio of approximately 4:1 and potential model instability. Model 3 and Model 4 were regarded as the primary analytical results. Statistical significance was assigned to two-sided P-values under 0.05, while those ranging from 0.05 to 0.1 indicated a trend.

## Results

### The study subjects’ characteristics

This study classified 1,058 inpatients with T2DM into four categories according to VFA quartiles: <46.3 cm², 46.3–72.7 cm², 72.8–101.7 cm², and >101.7 cm². Baseline characteristics by VFA quartiles are summarized in **Table 1**. An increase in VFA was associated with higher prevalence of dyslipidemia, SFA, SBP, DBP, BMI, FCP, C-peptide_120_min, TG, ALT, and SUA, and a decrease in HDL-C after controlling for age and sex (all P < 0.05). Significant differences were found across VFA quartiles in sex distribution, hypertension prevalence, and LDL-C levels (all P < 0.05), but not in diabetes duration, smoking or alcohol status, use of OADs, IIAs, or LLDs, nor in HbA1c, TC, FPG, glucose at 120 minutes, sCr or uACR levels.

**Table 1:** Characteristics of the subjects according to VFA.

Variables	Q1 (n=264)	Q2 (n=264)	Q3 (n=266)	Q4 (n=264)	P value	Adjusted P value
VFA (cm^2^)	<46.3	46.3-72.7	72.8-101.7	>101.7	—	—
Male (n, %)	135 (51.1%)	155 (58.7%)	151 (56.8%)	180 (68.2%)	<0.001	<0.001
Age (years)	58 ± 13	60 ± 13	60 ± 13	62 ± 13	<0.001	0.066
*DD (months)	78 (36-156)	84 (12-165)	100 (24-168)	84 (12-147)	0.310	0.547
Hypertension (n, %)	74 (28.0%)	116 (43.9%)	134 (50.4%)	108 (40.9%)	0.042	0.047
Dyslipidemia (n, %)	157 (59.5%)	162 (61.4%)	173 (65.0%)	191 (72.3%)	0.177	0.042
Smoking (n, %)	57 (21.6%)	60 (22.7%)	61 (22.9%)	56 (21.2%)	0.955	0.148
Alcohol (n, %)	33 (12.5%)	43 (16.3%)	36 (13.5%)	37 (14.0%)	0.852	0.447
IIAs (n, %)	67 (25.4%)	52 (19.7%)	57 (21.4%)	59 (22.3%)	0.520	0.513
LLDs (n, %)	55 (20.8%)	75 (28.4%)	64 (24.1%)	72 (27.3%)	0.257	0.221
OADs (n, %)	256 (97.0%)	245 (92.8%)	255 (95.9%)	240 (90.9%)	0.083	0.103
*SFA (cm^2^)	121.8 (100.4-156.0)	180.0 (154.4-209.2)	200.5 (166.1-232.5)	224.7 (191.4-290.3)	<0.001	<0.001
SBP (mmHg)	131 ± 19	134 ± 18	135 ± 16	139 ± 16	<0.001	<0.001
DBP (mmHg)	79 ± 11	81 ± 12	79 ± 10	84 ± 13	<0.001	<0.001
BMI (kg/m^2^)	22.97 ± 2.61	24.76 ± 2.49	26.68 ± 3.10	29.4 ± 4.08	<0.001	<0.001
HbA1c (%)	8.99 ± 2.54	9.09 ± 2.17	8.94 ± 2.03	8.81 ± 1.83	0.261	0.453
*TG (mmol/l)	1.20 (0.86-1.76)	1.72 (1.18-2.60)	1.72 (1.19-2.77)	1.98 (1.45-3.35)	<0.001	<0.001
TC (mmol/l)	4.47 ± 1.22	4.71 ± 1.32	4.59 ± 1.21	4.50 ± 1.5	0.358	0.171
HDL-C (mmol/l)	1.18 ± 0.31	1.08 ± 0.25	1.07 ± 0.27	1.02 ± 0.36	<0.001	<0.001
LDL-C (mmol/l)	2.58 ± 0.72	2.78 ± 0.67	2.79 ± 0.79	2.61 ± 0.79	0.034	0.012
*FPG (mmol/l)	7.84 (6.08-9.56)	7.97 (6.09-9.99)	8.10 (7.02-10.0)	8.13 (7.15-10.16)	0.360	0.395
*glucose_120_min (mmol/l)	17.30 (14.23-20.45)	17.36 (14.79-20.13)	18.01 (15.31-21.00)	18.07 (14.90-20.33)	0.737	0.906
*FCP (ng/mL)	1.61 (0.89-2.25)	1.83 (1.31-3.10)	2.05 (1.11-2.75)	2.95 (2.09-3.80)	<0.001	<0.001
*C-peptide_120_min (ng/mL)	4.57 (2.98-6.34)	4.93 (3.22-7.50)	5.62 (3.38-7.90)	6.21 (4.92-7.90)	<0.001	<0.001
*ALT (U/l)	16 (13-25)	20 (15-29)	20 (14-28)	25 (17-39)	<0.001	<0.001
*sCr (μmol/l)	65.3 (59.9-77.5)	73.3 (63.7-82.8)	75.4 (64.1-83.3)	72.8 (64.6-78.4)	0.087	0.892
*SUA (μmol/l)	277 (226-337)	329 (239-416)	334 (282-412)	362 (299-422)	<0.001	<0.001
uACR (mg/g)	59 ± 15	54 ± 15	63 ± 18	61 ± 17	0.419	0.445

Values are expressed as the mean ± S.D, or median with interquartile range, or percentages.

P value: The P-values were not adjusted for age and sex for the trend.

Adjusted P value: The P-values were adjusted for sex and age for the trend.

*The Kruskal-Wallis test was applied.

### Prevalence of visceral obesity in individuals with T2DM

**Figure 1:**
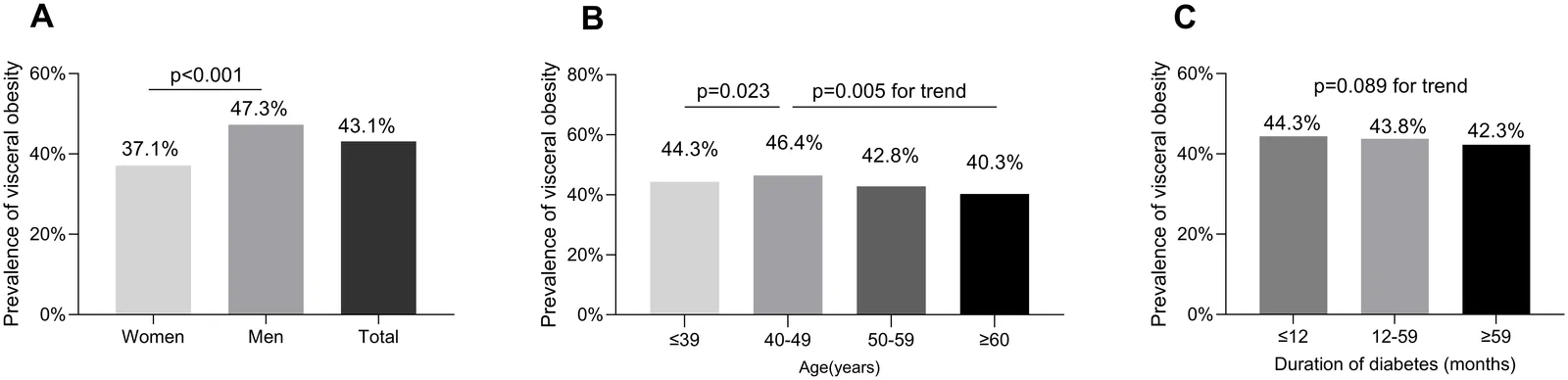
Prevalence of visceral obesity by gender, age, and DD groups. **(A)** Overall prevalence of visceral obesity, along with comparisons between gender-based groups. **(B)** Prevalence of visceral obesity compared across different age groups. **(C)** Comparisons of visceral obesity prevalence among patients stratified by DD.

The prevalence of visceral obesity is shown in **Figure 1** across various gender, age, and DD categories. Overall, 43.1% of the population had visceral obesity, with 47.3% of men and 37.1% of women affected. After adjusting for DD and age, men exhibited a higher prevalence than women (P < 0.001, **Figure 1A**). Among age groups, the highest rate of visceral obesity was observed in those aged 40–49 years (46.4%), with a decreasing trend in prevalence in individuals over 50 years (**Figure 1B**). Additionally, the prevalence of visceral obesity showed a decreasing trend as DD increased (**Figure 1C**).

### Ketosis prevalence comparisons in VFA quartile groups

**Figure 2:**
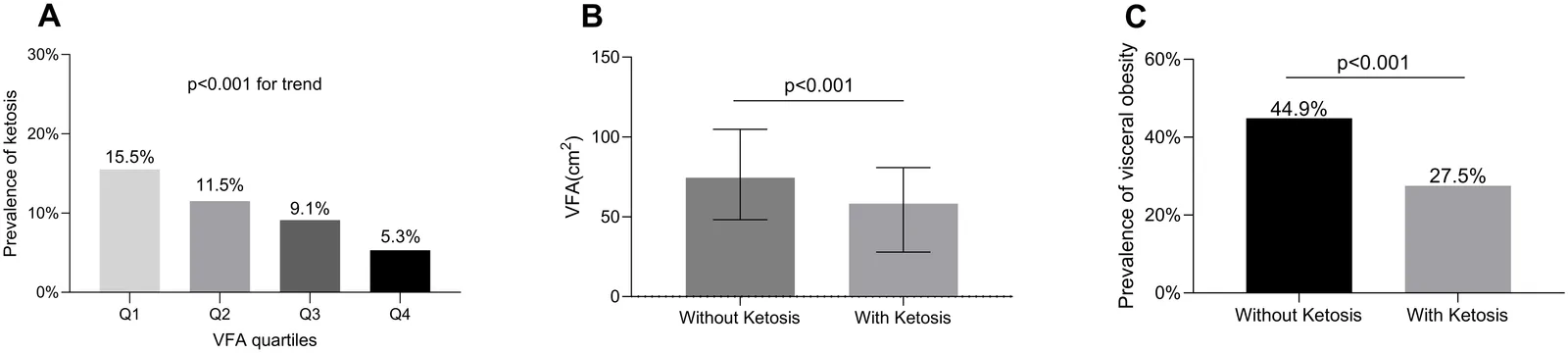
Prevalence of ketosis across VFA quartile groups. **(A)** Comparison of ketosis prevalence across different VFA quartile groups. **(B)** Differences in VFA between patients with and without ketosis. **(C)** Comparison of visceral obesity prevalence between patients with and without ketosis.

In **Figure 2**, we compare the prevalence of ketosis across different VFA quartiles and examine the link between VFA and the visceral obesity prevalence in T2DM patients with and without ketosis. The prevalence of ketosis was significantly lower in the second (11.5%), third (9.1%), and fourth (5.3%) VFA quartiles compared to the first quartile (15.5%), with the lowest prevalence observed in the fourth quartile after adjusting for age, gender, and DD (P < 0.001 for trend) (**Figure 2A**). Moreover, T2DM patients with ketosis had lower VFA values compared to those without ketosis (P < 0.001) (**Figure 2B**). Furthermore, T2DM patients with ketosis exhibited a lower rate of visceral obesity than those without ketosis (P < 0.001) (**Figure 2C**).

### Comparisons of MASLD prevalence, insulin secretion and glucose metabolism

**Figure 3:**
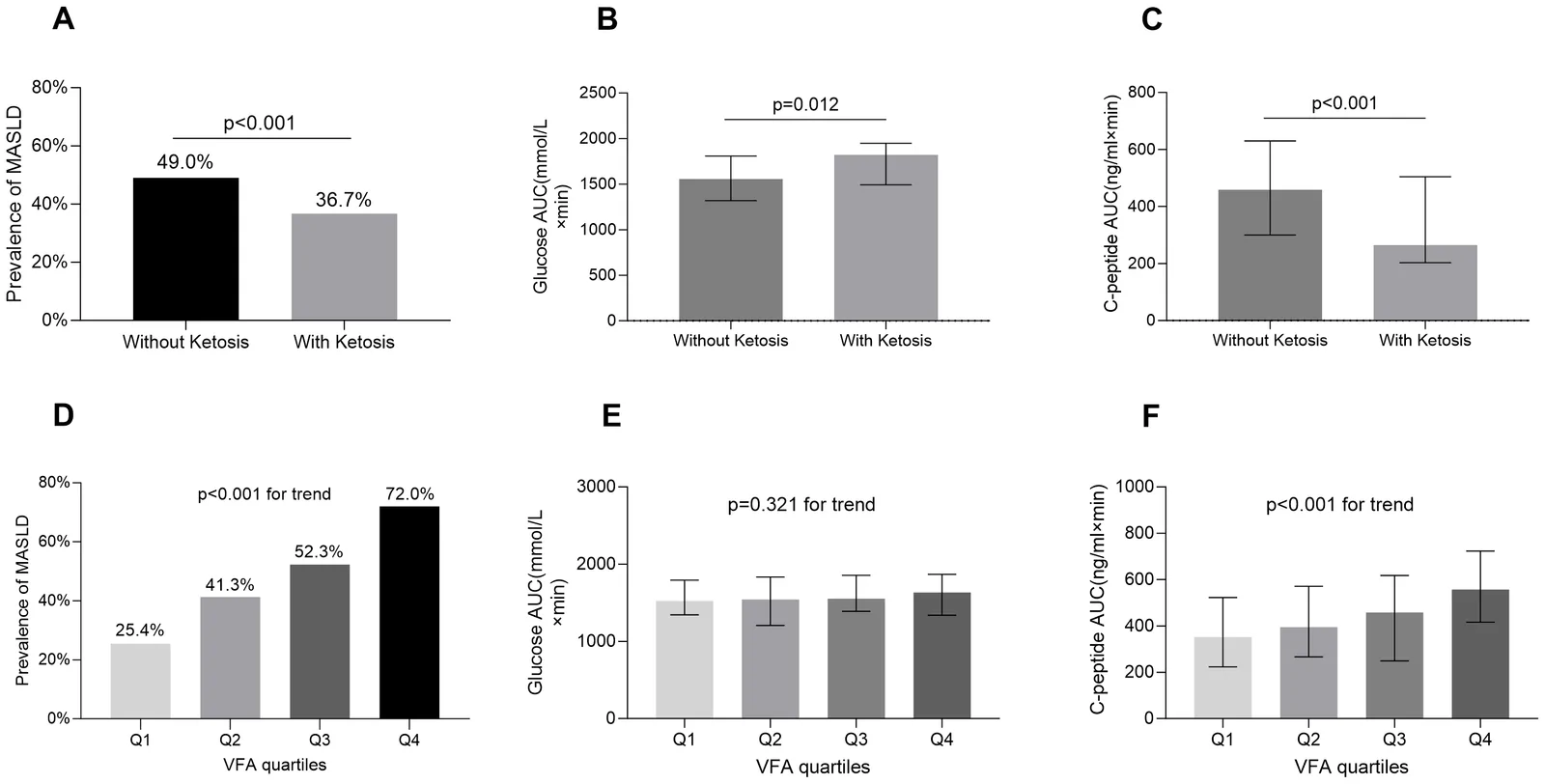
Prevalence of MASLD, insulin secretion, and glucose metabolism in relation to ketosis and VFA. **(A)** Comparison of MASLD prevalence between patients with and without ketosis. **(B)** Comparison of glucose AUC between patients with and without ketosis. **(C)** Comparison of C-peptide AUC between patients with and without ketosis. **(D)** Comparison of MASLD prevalence across VFA quartile groups. **(E)** Comparison of glucose AUC across VFA quartile groups. **(F)** Comparison of C-peptide AUC across VFA quartile groups.

**Figure 3** examines the prevalence of MASLD, glucose AUC, and C-peptide AUC in T2DM patients, comparing those with and without ketosis and across various VFA quartiles. After accounting for age, sex, and DD, the prevalence of MASLD and C-peptide AUC was lower, while glucose AUC was higher in T2DM patients with ketosis compared to those without ketosis (all P < 0.05, **Figures 3A–C**). The prevalence of MASLD progressively increased with higher VFA quartiles (P < 0.001 for trend, **Figure 3D**). A further comparison revealed that C-peptide AUC increased in the groups Q2, Q3, and Q4 compared to the group Q1 (P < 0.001 for trend, **Figure 3F**), while glucose AUC showed no significant difference across the Q1, Q2, Q3, and Q4 (P = 0.321 for trend, **Figure 3E**).

### Visceral obesity association with ketosis

**Table 2** presents binary logistic regression outcomes exploring the association between visceral obesity and ketosis. Significant inverse correlation was observed in the crude and preliminarily adjusted models (Model 1 and Model 2). Model 3 and Model 4 served as primary analyses, and both consistently confirmed that visceral obesity was independently linked to lower ketosis prevalence (all P<0.001). Model 5 was treated as a sensitivity analysis constrained by low events-per-variable ratio. Although statistical significance still existed (P = 0.026), its findings were interpreted with caution. As shown in **Supplementary Table 4**, all VIFs were <5.0, indicating no significant multicollinearity among covariates.

**Table 2:** Association of the prevalence of ketosis with visceral obesity.

Models	B statistic	OR	95% CI	*P* value
Model 1	-0.763	0.466	0.300-0.723	<0.001
Model 2	-0.877	0.416	0.260-0.666	<0.001
Model 3	-0.906	0.404	0.251-0.649	<0.001
Model 4	-0.895	0.409	0.251-0.644	<0.001
Model 5^a^	-0.714	0.490	0.261-0.919	0.026

Model 1: Unadjusted.

Model 2: Adjusted for age, sex, DD, smoking status, alcohol intake, dyslipidemia and hypertension.

Model 3: Further adjustment for use of LLD, IIAs, and OADs.

Model 4: Further adjustment for SBP, DBP, BMI, and SFA.

Model 5^a^: Sensitivity analysis with full additional adjustment for TC, TG, HDL-C, LDL-C, sCr, SUA, uACR, ALT, HbA1c, HOMA2-IR, glucose AUC, and C-peptide AUC. This model is limited by a low events-per-variable (EPV) ratio of approximately 4:1.

^a^ Model 3 and Model 4 are defined as the primary analytical results; Model 5a is presented as supplementary sensitivity analysis to verify the robustness of the association.

### Association of VFA quartiles with ketosis

**Table 3** illustrates the graded relationship between VFA stratification and ketosis occurrence. Higher ketosis prevalence among patients with lower VFA levels was observed in both unadjusted and initially adjusted analyses (Model 1 and Model 2). After further rigorous confounding adjustment, primary analyses based on Model 3 and Model 4 verified that ketosis rose gradually with the decline of visceral fat mass, with stable statistical significance (all P<0.05). The overall association trend remained consistent in Model 5 sensitivity analysis (P = 0.020).

**Table 3:** Association of the prevalence of ketosis with VFA quartiles.

	ORs (95% CI)				P values for trend
Models	Q4	Q1	Q2	Q3	
Model 1	1	3.270 (1.736-6.159)	2.300 (1.190-4.446)	1.771 (0.895-3.505)	0.002
Model 2	1	2.901 (1.453-5.789)	2.743 (1.351-5.568)	2.373 (1.145-4.919)	0.016
Model 3	1	2.812 (1.378-5.738)	2.793 (1.382-5.646)	2.396 (1.150-4.990)	0.019
Model 4	1	2.808 (1.370-5.753)	2.785 (1.340-5.788)	2.539 (1.210-5.325)	0.022
Model 5^a^	1	3.587 (1.402-9.179)	3.094 (1.298-7.376)	1.737 (0.660-4.571)	0.020

Model 1: Unadjusted.

Model 2: Adjusted for age, sex, DD, smoking status, alcohol intake, dyslipidemia and hypertension.

Model 3: Further adjustment for use of LLD, IIAs, and OADs.

Model 4: Further adjustment for SBP, DBP, BMI, and SFA.

Model 5^a^: Sensitivity analysis with full additional adjustment for TC, TG, HDL-C, LDL-C, sCr, SUA, uACR, ALT, HbA1c, HOMA2-IR, glucose AUC, and C-peptide AUC. This model is limited by a low events-per-variable (EPV) ratio of approximately 4:1.

^a^ Model 3 and Model 4 are defined as the primary analytical results; Model 5a is presented as supplementary sensitivity analysis to verify the robustness of the association.

### Sensitivity analysis

Sensitivity analyses excluding nine participants with DKA yielded results consistent with the primary findings. As shown in **Supplementary Table 1**, the inverse association between visceral obesity and ketosis prevalence remained statistically significant across all adjustment models (e.g., Model 3 adjusted OR: 0.366, 95% CI: 0.223–0.602; P<0.001). Similarly, when VFA was categorized into quartiles (**Supplementary Table 2**), participants in the lowest quartile (Q1) exhibited a markedly higher risk of ketosis compared with those in the highest quartile (Q4) (Model 3 OR: 3.968, 95% CI: 1.845–8.532; P for trend = 0.003). These findings confirmed that the observed associations were not attributable to individuals with DKA.

## Discussion

This study reveals that 47.3% of male patients with T2DM exhibit visceral obesity, indicating a higher tendency for abdominal fat accumulation in men compared to women. This observation is in line with prior research, which has demonstrated that male T2DM patients typically exhibit a higher average visceral fat mass compared to their female counterparts (28). Several factors may contribute to this discrepancy, including the larger size and greater quantity of chylomicrons in males, as well as increased dietary fat intake, which promotes the accumulation of visceral fat in abdominal adipocytes (29). Additionally, androgens may facilitate fat deposition in the abdominal region, while elevated social stress in males could lead to increased cortisol levels, further exacerbating the prevalence of visceral obesity in men (30, 31). Moreover, the current study identified that the prevalence of visceral obesity among patients with T2DM was most pronounced in individuals aged 40–49 years, surpassing that observed in those under 39 years of age. However, beyond the age of 50, there was a progressive decline in prevalence with increasing age. This pattern may be attributed to midlife weight gain, which is often reported due to a decrease in basal metabolic rate with age, coupled with factors such as reduced physical activity stemming from occupational and familial responsibilities, and declining estrogen levels in women, all of which contribute to central fat accumulation (32). As individuals continue to age, the prevalence of visceral obesity appears to decrease, potentially due to the onset of comorbidities, increased medication usage, diminished appetite, and a greater commitment to health-conscious lifestyle practices (33, 34). Furthermore, a longer DD was correlated with a decreasing trend in visceral obesity. Previous research has suggested that as diabetes progresses, some patients experience stabilization or even a reduction in waist circumference (35). This phenomenon may be attributed to lifestyle modifications and pharmacological interventions following diagnosis, which were associated with improvements in weight and fat distribution.

The association between obesity and ketosis in individuals with T2DM continues to be a topic of scholarly discussion. One study hypothesized that excessive visceral adiposity in T2DM patients may augment ketone production by altering fatty acid metabolism (36). Conversely, another study reported that T2DM patients experiencing ketosis exhibited a higher BMI compared to those without ketosis (37). However, prior research has demonstrated no significant correlation between BMI, medication use, and ketone levels in T2DM patients (21, 38). Furthermore, limited studies have directly examined the relationship between visceral fat and diabetic ketosis. This study seeks to investigate the connection between visceral fat and ketosis in T2DM patients to elucidate the relationship between visceral obesity and ketosis.

The results are consistent with a potential inverse association between visceral obesity and ketosis in patients with T2DM. Specifically, the presence of visceral obesity was associated with a more than 50% reduction in the likelihood of ketosis. As VFA quartiles decreased, there was a corresponding increase in the prevalence of ketosis among T2DM patients. Individuals with ketosis showed a lower rate of visceral obesity than those without ketosis. Supporting our results, a study involving approximately 200 T2DM patients found that the prevalence of obesity in those with ketosis was 20.6%, compared to 32.9% in non-ketotic T2DM patients, a statistically significant difference (20). Our analysis further revealed that the prevalence of ketosis was 1.79 times higher in Q2 group and 1.54 times higher in Q3 group compared to Q4 group. Although BMI alone does not classify T2DM patients as underweight, a VFA below 46.3 cm² is linked to a 1.81-fold increased prevalence of ketosis. Importantly, these associations remained robust after excluding participants with DKA and were consistently observed across both visceral obesity and categorical VFA analyses, supporting a dose–response relationship between visceral adiposity and ketosis susceptibility that is independent of conventional metabolic risk factors. Serum bicarbonate showed a positive trend across visceral fat quartiles in ketosis-positive participants (**Supplementary Table 3**), suggesting milder acid–base disturbance with higher visceral adiposity. These findings suggest that a specific level of visceral fat may be related to a decreased occurrence of ketosis in T2DM patients.

This study revealed a progressive increase in the prevalence of MASLD across higher quartiles of VFA. Aligning with our results, VFA was independently and positively correlated with the prevalence of MASLD, highlighting its role as a significant site for ectopic fat deposition (39). Additionally, our findings showed that patients with T2DM who went through ketosis had a lower occurrence of MASLD than those who did not. Similarly, a study involving 40 participants reported that as hepatic triglyceride levels increased, the production of ketone bodies was progressively impaired, resulting in decreased blood ketone levels (15). Ketone bodies serve as intermediate metabolites in the hepatic fatty acid oxidation process. Under normal physiological conditions, ketone body concentrations in the bloodstream are low. However, in the context of diabetes, where insulin deficiency hampers glucose utilization, fatty acids become the predominant energy substrate and undergo extensive oxidation in the liver, resulting in increased ketone body production. In patients with MASLD, hepatic lipid metabolism is altered, enhancing the activity of the tricarboxylic acid cycle. This enhancement reduces the conversion of acetyl-CoA into ketone bodies, as acetyl-CoA is preferentially directed towards oxidative metabolism and gluconeogenesis, thereby limiting ketone body synthesis (15, 40). Therefore, the increased prevalence of ketosis observed in the lower VFA quartiles may coincide with lower visceral fat levels and a reduced prevalence of MASLD. Alternatively, recurrent ketotic episodes could be associated with subsequent changes in fat distribution and hepatic metabolism, limiting ketone body synthesis.

The research identified a positive link between C-peptide AUC and higher quartiles of visceral fat, suggesting that serum insulin levels rise with more visceral fat. A prior study similarly identified a positive link between visceral fat and hyperinsulinemia in people with and without T2DM, regardless of BMI and subcutaneous fat (41). Furthermore, this study observed that T2DM patients experiencing ketosis exhibited lower insulin levels compared to those without ketosis, aligning with earlier research. A study on T2DM patients receiving SGLT2 inhibitor therapy found that reduced insulin levels increased the prevalence of ketosis (42). Another study indicated that pathological ketosis associated with uncontrolled diabetes and hyperglycemia arises from inadequate insulin production (43). Elevated insulin levels reduce ketone body synthesis by inhibiting the transport and β-oxidation of free fatty acids present in the liver (44). Consequently, among patients with T2DM and reduced visceral adiposity, lower insulin levels were observed alongside​ compromised glucose utilization, which may be temporally related to ketosis. However, given the cross-sectional design, whether reduced visceral fat precedes or follows ketotic episodes cannot be determined. In conclusion, lower visceral adiposity was cross-sectionally associated with a higher prevalence of ketosis, potentially reflecting underlying differences in insulin secretion and hepatic metabolism.

This study reveals that patients with T2DM and decreased visceral fat content face a significantly increased prevalence of ketosis. These results underscore several critical clinical considerations. Regular assessment of visceral fat in T2DM patients is crucial to identify those at higher prevalence for ketosis. Therapeutic interventions that increase ketosis should be carefully avoided in this patient group. Previous studies have indicated that although a low-carbohydrate diet alone poses a minimal risk of causing ketosis in T2DM patients, combining it with SGLT2 inhibitors significantly elevates this risk (45). Notably, our findings suggest that patients with low visceral fat may be more susceptible to ketosis. While this observation warrants clinical attention, direct recommendations regarding specific therapeutic interventions—including SGLT2 inhibitors or dietary modifications—cannot be made, as these factors were excluded from the current study. Future prospective studies are needed to evaluate whether tailored dietary counseling or glucose-lowering regimens can mitigate ketosis risk in this subgroup. This observation might illuminate the obesity paradox, where non-underweight T2DM patients frequently have better clinical outcomes than underweight patients, even though obesity is a significant risk factor for T2DM (46). Importantly, the cross-sectional design precludes causal inference. The observed inverse association between visceral fat and ketosis could plausibly reflect reverse causation, whereby recurrent ketotic episodes—rather than low visceral fat per se—contribute to subsequent changes in body composition, insulin secretion, or hepatic lipid metabolism. Longitudinal studies are required to clarify the temporal relationship between visceral adiposity and ketosis susceptibility. In this normal-BMI subgroup (Chinese criteria, <24 kg/m²), ketosis prevalence was highest in Q1 (16.8%) and absent in Q4 (0%), while visceral obesity (14.8%) showed a lower ketosis rate than non-visceral obesity (9.6% vs. 14.7%) (**Supplementary Table 5**). The skewed VFA distribution precluded further subgroup analyses. Larger adiposity-stratified studies are warranted.

This study has several limitations that should be acknowledged. First, visceral fat area was estimated by BIA rather than CT or MRI. Although BIA demonstrates favorable concordance with CT in Asian populations (correlation coefficient 0.917) (47), inherent measurement variability persists, particularly in patients with fluid disturbances, sarcopenia, or edema, which may affect bioimpedance signals. Second, ketosis was defined using urinary ketones measured by the nitroprusside method, which does not detect β-hydroxybutyrate—the predominant ketone body in insulin-deficient hyperglycemia. Hydration status and renal thresholds may further attenuate the agreement between urinary and serum ketone levels. Nevertheless, sensitivity analyses using alternative diagnostic thresholds verified the stability of our results. When urinary ketone ≥1+ was defined as ketosis, visceral obesity was negatively associated with diabetic ketosis (Model 4: OR = 0.519, 95%CI: 0.337–0.800, P = 0.003), and VFA quartile showed a marginally negative correlation (Model 4: OR = 0.809, 95%CI: 0.645–1.016, P = 0.068). Using urinary ketone ≥3+ as the cutoff, both visceral obesity (Model 4: OR = 0.342, 95%CI: 0.118–0.993, P = 0.049) and VFA quartile (Model 4: OR = 0.596, 95%CI: 0.361–0.983, P = 0.043) exerted significant negative correlations.​ These consistent inverse tendencies across different criteria support the robustness of our primary findings. Third, Model 5 was constrained by a low events-per-variable ratio, potentially introducing statistical instability; therefore, Models 3 and 4 were regarded as the primary analytical results.

Future studies should address these limitations by incorporating serum β-hydroxybutyrate measurements. Prospective longitudinal designs are warranted to establish temporal relationships and clarify whether low visceral fat predisposes to ketosis or results from recurrent ketotic episodes. Additionally, multicenter studies with larger sample sizes are needed to validate this association across diverse BMI strata, particularly within the normal-weight subgroup. Mechanistic investigations into hepatic lipid metabolism and insulin secretion dynamics may further elucidate the biological pathways linking visceral adiposity to ketogenesis.

## Conclusion

This study suggests that lower visceral fat mass may be potentially associated with elevated ketosis in patients with type 2 diabetes. Assessment of visceral fat status may help inform individualized dietary and pharmacological management. Clinicians should pay close attention to ketosis among patients with low visceral fat.

## Data Availability

The raw data supporting the conclusions of this article will be made available by the authors, without undue reservation.
