# Supplementary material for: Higher visceral fat area is inversely associated with ketosis in Chinese patients with type 2 diabetes mellitus: a cross-sectional study
Source: Front Endocrinol (Lausanne). 2026 Sep 11;17:1948929. doi: 10.3389/fendo.2026.1948929 (PMC13612281; doi:10.3389/fendo.2026.1948929)
Supplement: Supplementary file 1 [file Table1.docx]

**Table** **S1. Association between ketosis prevalence and visceral obesity in patients without diabetic ketoacidosis (DKA)**

|  | B statistic | OR | 95% CI | P value |
| --- | --- | --- | --- | --- |
| Model 1 | -0.856 | 0.425 | 0.267-0.676 | <0.001 |
| Model 2 | -0.920 | 0.399 | 0.244-0.651 | <0.001 |
| Model 3 | -1.005 | 0.366 | 0.223-0.602 | <0.001 |
| Model 4 | -0.645 | 0.525 | 0.300-0.918 | 0.024 |
| Model 5^a^ | -0.634 | 0.530 | 0.295-0.953 | 0.034 |

Model 1: Unadjusted.

Model 2: Adjusted for age, sex, DD, smoking status, alcohol intake, dyslipidemia and hypertension.

Model 3: Further adjustment for use of LLD, IIAs, and OADs.

Model 4: Further adjustment for SBP, DBP, BMI, and SFA.

Model 5^a^: Sensitivity analysis with full additional adjustment for TC, TG, HDL-C, LDL-C, sCr, SUA, uACR, ALT, HbA1c, HOMA2-IR, glucose AUC, and C-peptide AUC. This model is limited by a low events-per-variable (EPV) ratio of approximately 4:1.

^a^ Model 3 and Model 4 are defined as the primary analytical results; Model 5^a^ is presented as supplementary sensitivity analysis to verify the robustness of the association.
