# Supplementary material for: Higher visceral fat area is inversely associated with ketosis in Chinese patients with type 2 diabetes mellitus: a cross-sectional study
Source: Front Endocrinol (Lausanne). 2026 Sep 11;17:1948929. doi: 10.3389/fendo.2026.1948929 (PMC13612281; doi:10.3389/fendo.2026.1948929)
Supplement: Supplementary file 2 [file Table2.docx]

**Table** **S2. Association of the prevalence of ketosis with VFA quartiles without diabetic ketoacidosis (DKA)**

|  | ORs (95% CI) | | | | P values for trend |
| --- | --- | --- | --- | --- | --- |
|  | Q4 | Q1 | Q2 | Q3 |  |
| Model 1 | 1 | 4.140(2.013-8.515) | 3.256(1.558-6.807) | 2.519(1.180-5.378) | 0.001 |
| Model 2 | 1 | 4.273(2.026-9.014) | 4.047(1.872-8.750) | 3.466(1.564-7.683) | 0.001 |
| Model 3 | 1 | 3.968(1.845-8.532) | 3.879(1.788-8.414) | 3.374(1.523-7.476) | 0.003 |
| Model 4 | 1 | 2.952(1.288-6.764) | 2.812(1.210-6.534) | 2.513(0.962-6.563) | 0.066 |
| Model 5^a^ | 1 | 3.559(1.430-8.857) | 3.717(1.542-8.961) | 2.992(1.180-7.587) | 0.024 |

Model 1: Unadjusted crude model.

Model 2: Adjusted for age, sex, DD, smoking status, alcohol intake, dyslipidemia and hypertension.

Model 3: Further adjusted for LLDs, IIAs, and OADs.

Model 4: Further adjusted for SBP, DBP, BMI, and SFA.

Model 5^a^: Sensitivity analysis with full additional adjustment for TC, TG, HDL-C, LDL-C, sCr, SUA, uACR, ALT, HbA1c, HOMA2-IR, glucose AUC, and C-peptide AUC. This model is limited by a low events-per-variable (EPV) ratio of approximately 4:1.

^a^ Model 3 and Model 4 are defined as the primary analytical results; Model 5 is presented as a supplementary sensitivity analysis only, and its results are interpreted cautiously due to potential instability.
