# Supplementary material for: Higher visceral fat area is inversely associated with ketosis in Chinese patients with type 2 diabetes mellitus: a cross-sectional study
Source: Front Endocrinol (Lausanne). 2026 Sep 11;17:1948929. doi: 10.3389/fendo.2026.1948929 (PMC13612281; doi:10.3389/fendo.2026.1948929)
Supplement: Supplementary file 3 [file Table3.docx]

| VFA quartile | n | HCO₃⁻ (mmol/L) | P | P* |
| --- | --- | --- | --- | --- |
| Q1 (<46.3 cm²) | 41 | 21.9 ± 2.9 | ref | ref |
| Q2 (46.3-72.7 cm²) | 30 | 22.3 ± 2.8 | 0.53 | 0.48 |
| Q3 (72.8-101.7 cm²) | 24 | 23.6 ± 2.6 | 0.083 | 0.06 |
| Q4 (>101.7cm²) | 14 | 24.5 ± 2.5 | 0.015 | 0.012 |
| P for trend |  |  | 0.011 | <0.001 |

**Table S3. Serum bicarbonate levels stratified by visceral fat area quartiles in participants with ketosis**

P vs. Q1 (Tukey's post-hoc); *Adjusted for age, sex
