# Supplementary material for: Higher visceral fat area is inversely associated with ketosis in Chinese patients with type 2 diabetes mellitus: a cross-sectional study
Source: Front Endocrinol (Lausanne). 2026 Sep 11;17:1948929. doi: 10.3389/fendo.2026.1948929 (PMC13612281; doi:10.3389/fendo.2026.1948929)
Supplement: Supplementary file 4 [file Table4.docx]

| Variables | Tolerance | VIF |
| --- | --- | --- |
| Age | 0.808 | 1.238 |
| Sex | 0.812 | 1.232 |
| Diabetes duration | 0.65 | 1.538 |
| Smoking | 0.532 | 1.88 |
| Alcohol | 0.595 | 1.682 |
| Hypertension | 0.585 | 1.709 |
| Dyslipidemia | 0.58 | 1.724 |
| OADs | 0.819 | 1.222 |
| IIAs | 0.739 | 1.354 |
| LLDs | 0.909 | 1.1 |
| SBP | 0.609 | 1.643 |
| DBP | 0.61 | 1.64 |
| SFA | 0.789 | 1.267 |
| VFA | 0.450 | 2.224 |
| BMI | 0.387 | 2.586 |
| HOMA-IR | 0.63 | 1.587 |
| Glucose AUC | 0.772 | 1.296 |
| C-peptide AUC | 0.559 | 1.788 |
| uACR | 0.788 | 1.27 |
| HbA1c | 0.748 | 1.338 |
| ALT | 0.755 | 1.324 |
| TC | 0.551 | 1.813 |
| TG | 0.722 | 1.385 |
| HDL | 0.409 | 2.447 |
| LDL | 0.292 | 3.430 |
| sCr | 0.823 | 1.215 |
| SUA | 0.78 | 1.281 |

Table S4. Variance inflation factors (VIF) and tolerance values for all covariates.

Variance inflation factors (VIFs) were derived using linear regression diagnostics with identical covariate sets. A VIF < 5.0 was considered indicative of acceptable independence among predictors.
