# Supplementary material for: Higher visceral fat area is inversely associated with ketosis in Chinese patients with type 2 diabetes mellitus: a cross-sectional study
Source: Front Endocrinol (Lausanne). 2026 Sep 11;17:1948929. doi: 10.3389/fendo.2026.1948929 (PMC13612281; doi:10.3389/fendo.2026.1948929)
Supplement: Supplementary file 5 [file Table5.docx]

| Variable | Category | Total n (%) | Ketosis present, n (%) | Ketosis absent, n (%) |
| --- | --- | --- | --- | --- |
| Visceral obesity | No (VFA <80 cm²) | 300 (85.2) | 44 (14.7) | 256 (85.3) |
|  | Yes (VFA ≥80 cm²) | 52 (14.8) | 5 (9.6) | 47 (90.4) |
| VFA quartiles | Q1 (lowest) | 184 (52.3) | 31 (16.8) | 153 (83.2) |
|  | Q2 | 101 (28.7) | 11 (10.9) | 90 (89.1) |
|  | Q3 | 52 (14.8) | 7 (13.5) | 45 (86.5) |
|  | Q4 (highest) | 15 (4.3) | 0 (0.0) | 15 (100.0) |
| Total | — | 352 (100) | 49 (13.9) | 303 (86.1) |

**Table S5. Distribution of visceral adiposity and ketosis prevalence in the normal-BMI subgroup.**
